# Automated License Plate Recognition for Resource-Constrained Environments

**DOI:** 10.3390/s22041434

**Published:** 2022-02-13

**Authors:** Heshan Padmasiri, Jithmi Shashirangana, Dulani Meedeniya, Omer Rana, Charith Perera

**Affiliations:** 1 Department of Computer Science and Engineering, University of Moratuwa, Moratuwa 10400, Sri Lanka; heshanpadmasiri.16@cse.mrt.ac.lk (H.P.); aajithmishashirangana.16@cse.mrt.ac.lk (J.S.); dulanim@cse.mrt.ac.lk (D.M.); 2 School of Computer Science and Informatics, Cardiff University, Cardiff CF10 3AT, UK; ranaof@cardiff.ac.uk

**Keywords:** edge computing, resource-constrained devices, energy efficiency, low cost, night vision

## Abstract

The incorporation of deep-learning techniques in embedded systems has enhanced the capabilities of edge computing to a great extent. However, most of these solutions rely on high-end hardware and often require a high processing capacity, which cannot be achieved with resource-constrained edge computing. This study presents a novel approach and a proof of concept for a hardware-efficient automated license plate recognition system for a constrained environment with limited resources. The proposed solution is purely implemented for low-resource edge devices and performed well for extreme illumination changes such as day and nighttime. The generalisability of the proposed models has been achieved using a novel set of neural networks for different hardware configurations based on the computational capabilities and low cost. The accuracy, energy efficiency, communication, and computational latency of the proposed models are validated using different license plate datasets in the daytime and nighttime and in real time. Meanwhile, the results obtained from the proposed study have shown competitive performance to the state-of-the-art server-grade hardware solutions as well.

## 1. Introduction

The emergence of edge computing has unveiled an exceptional proliferation of computer-intensive applications for smart cities [1,2] and smart homes [3] for different domains such as security [4], city parking [5] and traffic management [6]. Most of these modern systems involve capabilities beyond traditional computing by embedding edge intelligence to enable self-learning solutions including machine learning and deep learning [7,8,9]. Generally, edge-based solutions tend to be reliable and efficient due to the associated on-device decision-making and data-computing inclinations. However, edge computing inherits a new set of challenges in terms of resource management, data accumulation, and energy consumption [10,11]. As opposed to traditional internet-of-things (IoT) networks, edge computing minimizes the network load, thus reducing system latency. For instance, real-time applications such as vehicle license plate identification in smart cities usually have higher latency values [9]. However, with edge computing technology, these can be processed at the edge without sending the data to a central cloud [10,11]. Hence, it is increasingly important to put basic timely computations approximate to the physical system, as it reduces the latency of the overall system by multiple times.

This paper proposes an Automated License Plate Recognition (ALPR) solution for edge computing with resource-constrained environments, which can lead to support smart city development and management processes. Although ALPR is a well-established area in the domain of image processing, research on ALPR is still challenging with the associated constraints in the environment such as varying weather conditions, plate variations across regions, vehicle motion, distorted characters, dirty plates, shadow and reflection [9]. Moreover, most of the existing ALPR solutions limited to execution in server-grade hardware with nearly unlimited resources and limited to daytime performance. Thus, currently, there has been less attention paid to build systems that work efficiently in constrained environments targeting low cost, energy efficiency, less computational power requirements, remote location deployments and work in night vision. The technological developments of deep-learning techniques can be improved to use in edge devices to provide an efficient solution for ALPR in resource-constrained environments.

We present an approach and a proof-of-concept prototype for hardware-efficient ALPR at nighttime, while adhering to several constraints in terms of energy efficiency, resource use, low cost, low-latency communication and computation as the novel contributions. The proposed ALPR system can operate at nighttime without any visible additional illumination and require no Internet connection for operation. Consequently, the system is fully implementable on low-power edge devices such as Raspberry Pi 3b+ and operated completely with a battery that lasts long due to the energy-saving strategies implemented in the solution. Therefore, the system recognizes license plates in real time both day and nighttime, and can be deployed in rural or forest areas, where there is no stable Internet connectivity or a direct power grid, which is one of the main contributions of this study.

Our methodology uses deep-learning-based Neural Architecture Search (NAS) strategies to discover a novel set of hardware-efficient neural networks for autonomous management of license plate detection and recognition process for edge devices with low resources. The proposed differentiable architecture search is based on FB-Net (Facebook-Berkeley-Nets) [12] and PC-DARTS (Partially Connected Differentiable architecture search) [13]. These algorithms seek effective architectures without comprising the performance, by sampling a small part of a super-network to reduce the redundancy in exploring the network space. Thus, compared to the general approaches such as reinforcement learning, and evolutionary algorithms, the differentiable architecture search proposed in this study provides a significant reduction in computational power required to search neural networks.

Although neural networks for license plate recognition is a well-explored area for the daytime images with the server-grade hardware specification, we provide a solution for ALPR with limited resources in constraint environments. Moreover, compared to the existing studies as stated in Table 1, to the best of our knowledge, we provide a novel contribution to design and develop models to detect and recognize license plates using low-resource edge devices with different configurations. Thus, the implementation of the NAS-based data engineering techniques in IoT applications for hardware-efficient ALPR solutions, is one of the scientific contributions of this study. Therefore, the main focus of this study was to design and develop neural network-based models that are competitive with state-of-the-art models such as RPNet (Roadside Parking Net) [14] that are designed for server-grade hardware, consumes more memory, and are computationally expensive to execute on edge devices.

However, it is challenging to train the discovered deep neural networks to recognize license plates due to the lack of a large, annotated and diverse dataset. To circumvent this issue, we use a synthetic data generation process based on image-to-image translation techniques to convert daytime RGB (Red–Green–Blue) images into thermal infrared (TIR) images. The presented data synthesising process is inspired by the related work that has shown promising results in license plate recognition, as given in Table 2. Thus, we provide synthetic data generation approaches to mitigate the issue with the scarcity of a large and diverse nighttime license plate data set for the learning process of deep-learning models. Accordingly, this study uses 200,000 daytime license plate images from the CCPD data set Chinese City Parking Data set (CCPD) [14], and the corresponding nighttime images generated synthetically. Additionally, we use 100 nighttime images captured in a real environment showing the possibility of using the proposed approach for different other license plate data sets.

The prototype of our solution simulates a case study of an animal poacher vehicle detection problem. At present, Wildlife has faced a capacious and prejudicial issue that has caused a countable number of wild animals to lose their lives. Most of the existing approaches to minimize illegal hunting of wild animals, rely on manual surveillance from the camera feeds. Poacher vehicle detection system uses modern image processing and deep-learning techniques to detect poacher vehicles while tracking their license plate numbers and sending the detected vehicle details to authorized parties through SMS. It has been noticed that poachers arrive mostly at nighttime since the poacher vehicle detection system is designed to function at nighttime as well. The case study environment contains several constraints. This system relies on battery power only, thus the power consumption should be minimized. Since there is no Internet connectivity in the wild, SMS is the only possible communication method, where images can be stored for later prosecution material. Additionally, the system should be deployed in an unnoticeable way to the poachers. Thus, the proposed ALPR solution considers the following requirements.

- The system executes autonomously in real time on an edge platform with constrained memory and computational capabilities.
- The system is feasible, low cost and energy efficient to be deployed in the wild or remote areas, where there is no reliable Internet connection or a power grid.
- The system operates at nighttime without additional lighting that is visible to the naked eye.

Furthermore, the solution we present can be used to develop smart city-based applications such as identifying fraudulent vehicles and overcome security challenges with low resources in a cost-effective way. Thus, supports energy-efficient and low-latency communication and computation. Therefore, the novel approach we proposed directs towards the future perspective in edge computing.

The rest of the article is organized as follows. Section 2 reviews the literature in the field of automatic license plate recognition systems in embedded platforms and high-end serve-grade hardware. The design overview of the proposed solution is presented in Section 3. Section 4 analyses the results, and Section 5 discusses the findings. Section 6 concludes the study.

## 2. Background and Related Studies

### 2.1. Overview of LP Recognition Approaches

Over the time, many research studies have addressed Automated License Plate Recognition (ALPR). Yet, most of these solutions are designed to be executed on server-grade hardware with sufficient resources. In early stages of ALPR domain, most of the studies applied well-defined traditional computer vision techniques such as edge detection [36,37,38,39], genetic algorithms [40], and fuzzy logic [33] for both license plate detection and recognition. Although these solutions were faster, simple, and lightweight, they still lacked better performance when complex scenarios are involved. These techniques were often sensitive to noise, illumination variations and were mostly unable to place the license plates when they are inclined or deformed.

However, with the development of data engineering techniques, researchers have considered machine learning and deep-learning-based solutions for ALPR [30,41,42,43] with the aim of achieving high performance than the prevailing traditional solutions. However, these solutions consume more resources and processing power when compared to classical methods. In deep learning, the problem of automatic license plate recognition was considered to be a general object detection and a character recognition problem. Therefore, some researchers [44,45] used generic object detection models such as YOLO [46] to detect the license plate. However, these methods were more robust to noise, illuminations and inclinations of the plates thus eliminating most of the limitations in the classical methods.

### 2.2. LP Recognition in Constrained Environment

Computer vision applications are often developed to replace human in harsh, dangerous or tedious situations to handle numerous applications. Such harsh environments often raise many challenging conditions which are hard to tackle in naive ways. Among them, night vision is a pivotal area in most of the safety-critical applications such as surveillance, automotive safety [47], military defence systems [48]. Traditionally, there are common ways to capture nighttime images such as low-light-level (image-intensified) cameras, and thermal infrared (TIR) cameras. Nevertheless, the widely used approach in most modern applications is thermal imaging. These thermal images are sensitive to the infrared region of the electromagnetic spectrum, and they use variations in the temperature levels of the objects and the background to distinguish the objects in a TIR image. The main advantage of using TIR images is that they are robust against any illumination variations and can also be used to capture images at nighttime in complete darkness. They also produce quality images with no or few distortions during difficult weather conditions. However, thermal cameras are quite costly, and the scarcity of TIR datasets limits most of its applications. Therefore, a practical solution to mitigate this issue is to convert the available RGB (Red–Green–Blue) image datasets to TIR images.

A systematic study of converting RGB images to TIR was reported by Zhang et al. [49]. A large set of synthetic data generated by this work has provided accurate results than a small dataset with real TIR images in the field of object tracking. They have shown that a combination of real TIR images and the generated synthetic data gives the best results while tracking objects. They have used mainly two image-to-image translation methods called pix2pix [50] and cycleGAN [51]. Moreover, some applications use filters such as grey-scaling to transform daytime images to night-vision images. In another related study, Ismail et al. [52] have used an effective object detection method called Cascade classifier to function at nighttime and rainy weather conditions. They have enhanced the images using the top-hat transform operation. Another novel feature-based algorithm has presented in [53] to localize license plates even in complex situations such as different illumination and weather conditions. They have used an edge-based approach based on vertical edges and morphological operations. This study has shown an accuracy of 96.5% and has created a database with 269 images in challenging environments. Multiple-intensity IR-illuminator-based license plate detection in the nighttime has presented in [54]. Although infrared light allows detecting license plates under different illuminations, it does not perform well, when the distance from the target is changing. The authors have addressed this issue using a multiple-intensity IR illuminator that detects license plates at different levels of illuminations and distances and showed an accuracy of 98%.

Furthermore, except for changing illuminations, some hazardous weather conditions such as rain, fog, snow have always made the license plate recognition problem complex. However, few ALPR models are robust to these challenging situations in outside uncontrolled environments. Azam and Islam [55] have proposed such an ALPR algorithm to process license plates in rainy and foggy weather by removing rain streams and fog from the images captured. Accordingly, the complexity of the license plate detection task is greatly influenced by different environmental conditions. Although many studies have addressed license plate detection and recognition, only a few can be applied to an uncontrolled complex situation such as nighttime illumination, and extreme weather conditions [9]. In another point of view, even though the retro-reflective nature of license plates makes them readable even at night, still, it is challenging to accurately locate a license plate at nighttime, for reasons such as the insufficient amount of light to acquire the details. The use of an illuminator can be used to solve this issue to some extent. In addition, the emission of too much light from headlights also causes difficulty in reading license plates, as the plate reflects more light and the resulted brightness makes it hard to extract the data on the license plate. Thus, the related applications with computer vision techniques face challenges in situations such as changing weather conditions, issues with camera and equipment, moving object detection, demand for excessive resources and power.

### 2.3. ALPR Using Edge Devices

Edge computing enables offloading computational tasks to perform at the edge devices in contrast to the traditional social sensing approaches [56]. With the growth of data being produced at edge devices, it is becoming increasingly difficult to carry out all the necessary computations in the cloud with an acceptable latency. Edge computing supports solves this issue by merely increasing the computational capabilities of the edge devices, thus reducing the communication cost and the application latency. Moreover, it has become possible to due to the increase in computational performance in edge devices without significantly compromising energy efficiency [57].

Another reason to increase the computational capabilities in edge devices is the development of hardware accelerators for edge devices. These are dedicated hardware components such as Graphical Processing Units (GPUs) that enhance the graphical performance of the computer and Tensor Processing Units (TPUs) that accelerate application-specific integrated circuit (ASIC) and are used to improve performance in certain parts of programs thus lessen the execution time for deep neural networks. Such accelerators had been used in large servers in the cloud environment for a relatively long time. However, large energy efficiency can be achieved on edge devices by applying these accelerators, as it produces a large increase in the rate of computation for every watt of power consumed.

Data processing within edge devices, without moving computational loads for cloud services, has clear advantages. For instance, Hochstetler et al. [58] have shown that a neural network can be speedup by a factor of 1137% by adding an Intel^®^ Movidius^TM^ Neural Compute Stick (NCS), which is an accelerator that draws a maximum of 2.5 W of power to a Raspberry Pi 3B that has a maximum power draw of 6.7 W execution of MobileNet [59]. That is a large performance increase compared to a power increase of less than 40%. Such accelerators allow the execution of computations that would otherwise require cloud servers on edge devices. Moreover, Yi et al. [60] and Ha et al. [61] have demonstrated the improvements in response time by shifting computations to the edge devices. Additionally, by minimizing the amount of data that needs to be transmitted, Chun et al. [62] have shown up to 40% improvement in energy consumption can be achieved by shifting to edge computing.

In a related study of license plate recognition on embedded systems, Lee et al. [15] have proposed an ALPR system to detect Korean license plates on an NVIDIA Jetson TX1 embedded board. They have used a simple convolutional neural network (CNN) architecture called “AlexNet” and claimed a high recognition accuracy of 95.24%, but on a small dataset with 63 input images. Another study by Luo et al. [63] have designed a low-cost, high-speed, real-time embedded ALPR system based on a Digital Signal Processor (DSP). In this solution, they have ensembled a variety of peripheral modules to fulfil several requirements such as memory, input image acquisition, and networking etc. Nevertheless, the proposed solution is claimed to consume less power, high speed and precise enough to perform real-time license plate recognition in practical applications. Rezvi et al. [17] have proposed another solution to detect Italian license plates on a mobile platform by simplifying the architectures of two different pre-trained CNNs for license plate detection and recognition. However, this simplification flow introduces a trade-off between the accuracy and the execution time. Thus, a decrease in accuracy is expected regarding the network simplification process. Moreover, they have examined the system on two different GPU environments, such that a desktop workstation equipped with a Quadro K2200 GPU card and a powerful Jetson TX1 embedded board. In both environments, the simplified networks show lesser execution time than the original networks. Additionally, by converting the trainable parameters from double to float, they have reduced the memory consumption of both plate and character classifiers by half. However, this indeed has reduced the accuracy of the simplified architectures when compared to the original networks.

Accordingly, many solutions for license plate detection and recognition have been discussed extensively in the literature [9]. Most of the prevailing solutions in the domain of ALPR have addressed unrestricted environments such as a desktop computer with powerful processors. These solutions are designed to achieve maximum accuracy while assuming the availability of sufficient computational resources. However, this assumption does not valid for edge devices such as Raspberry Pi. Such environments often demand a small model with low complexity and low-resolution input images. One likely explanation for the low popularity of license plate detection and recognition solutions on the edge is the difficultly of handling the complexity of the computations in the limited resources in the edge devices. Furthermore, these ALPR solutions are expected to be effective and efficient to satisfy the real-time constraints of an embedded platform.

Table 1 states a summary of the selected existing edge-based solutions for license plate recognition with daytime (D), nighttime(N) and synthesised (S) data. Most of the related studies have been implemented on modern hardware settings, and may not execute on edge devices with limited resources. They were tested on powerful machines with powerful GPUs [15,17,25,26]. In addition, a few studies have provided solutions for embedded platforms with low resources [18]. Although the accuracies of the proposed models do not outperform the existing server-grade models such as RPNet [14] and TE2E [64] that require powerful GPUs, our aim of this study is to show the competing results of the proposed models that can be run on edge devices with limited resources. At the same time, the presented mid-tier and high-tier models show superior performance to license plate detection using Yolo-V3 [31,65]. This shows that our models are competitive with the existing state-of-the-art solutions in terms of accuracy.

Moreover, most of the studies have considered only daytime images [15,16] and only a few studies have considered nighttime and synthesised data [17]. Considering the challenges and limitations in the existing studies, we present a family of models based on NAS are designed for different hardware tiers of edge devices, in a way that the complexities of the proposed models are relatively low compared to server-grade models. Our solution can execute entirely on edge devices such as Raspberry Pi with limited memory and power constraints, showing competing results as stated in Section 4.3. Additionally, our solution has been tested for both daytime, synthetic, real nighttime data, and shown the best accuracies of 99.87%, 94.%, 98.82%, respectively.

In our previous study [57], we have discussed the architecture of the Lite LP-Net models in detail. As the next phase, this paper mainly describes the hardware circuit configurations from the deployment point of view, synthetic data generation process, stochastic super-network implementation and the bi-level optimization in Section 3, as the scientific contribution.

### 2.4. ALPR with Synthetic and Nighttime Images

Several studies have used the synthesised image for both daytime and nighttime license plate recognition with promising results. Table 2 shows the existing studies that have used nighttime (NT) and synthetic (Syn.) images. The performance metrics include accuracy (AC), false negative (FN), recall (R), average precision (AP) and F-score (F). Most of these studies were implemented on server-grade hardware settings. The study by Wu et al. [20], have achieved accuracy improvement using synthetic data and fine-tuning with a limited number of real data. However, the results depend on many factors such as the type of the dataset, optimization methods and used hyperparameters in deep-learning-based models. In [23], the best performing models have a large ratio of synthesised data using techniques such as CycleGAN, which strengthens the usefulness of the approach. In this study, our data synthesising method is inspired by the Generative Adversarial Network (GAN) and we used GAN-based pix2pix [20,49], as describe in Section 3.2. Moreover, several studies have used nighttime images in LP recognition. Considering the performance values, it can be observed that synthesised nighttime images have shown better results as well. However, they were not focused on implementation with low-resource settings, as we have considered in this study.

## 3. System Design and Methodology

### 3.1. Design Aspects of the Proposed ALPR System

**Figure 1:**
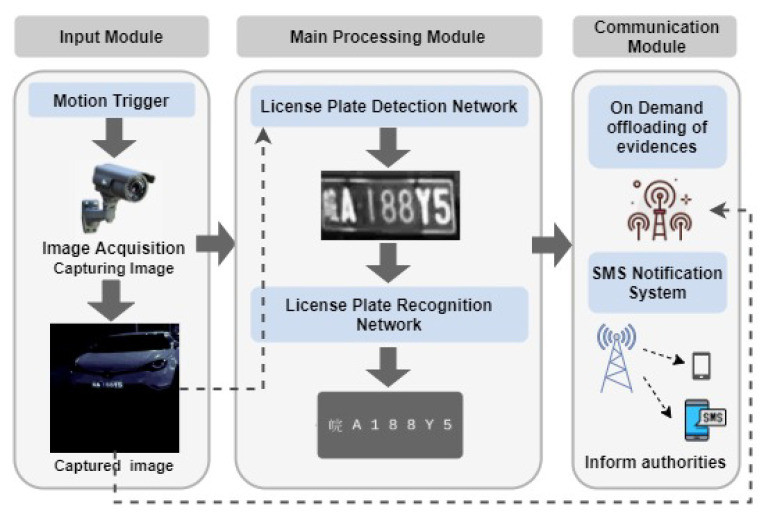
Overview of the proposed model.

**Figure 2:**
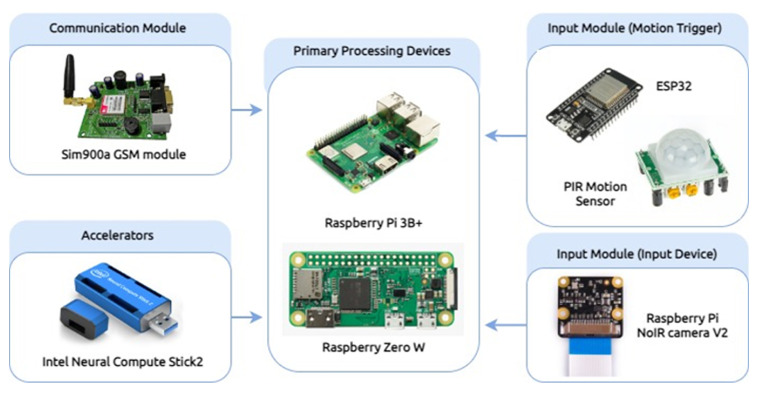
Hardware stack of the proposed solution.

The proposed system design consists of three main modules: input module, main processing module, and communication module as shown in Figure 1. The input module captures the vehicle images and feed them to the main processing module. Meanwhile, the main processing module performs the core functions of the system, which are license plate detection and recognition. Upon the retrieval of results from the license plate recognition stage, the communication module handles the data communication between the ALPR system and its operators. Figure 2 shows the hardware stack of our solution. The corresponding hardware specifications are given in Section 3.1.1.

#### 3.1.1. Cost-Effective Mobile-Sensing Data Communication Specifications

##### Raspberry Pi 3 Model B+

We used Raspberry Pi 3 Model B+, which is a well-balanced single-board computer as the default low-cost edge platform since it represents the middle ground of most of the product solutions. It can execute deep-learning models while being both relatively inexpensive and power-efficient, with 4 Cortex-A53 64-bit cores clocked at 1.4 GHz and 1 GB of LPDDR2 RAM [66]. Although the original Model B supports Bluetooth 4.1, B+ also advances its support for Bluetooth 4.2. The Model B+ also has a dual-band wireless antenna, supporting 2.4 GHz and 5 GHz 802.11 b/g/n/ac Wi-Fi.

##### Raspberry Pi Zero

We also used Raspberry Pi Zero, which consists of 1 GHz single-core processor and 512 MB of RAM [66]. Although the Raspberry Pi Zero model is not as powerful as the Raspberry Pi 3 Model, it is cheaper, power-efficient and smaller in model size than the Raspberry Pi 3. Thus, Raspberry Pi Zero is used as an edge platform for situations, where the Raspberry Pi 3 is expensive or consumes more power. However, with comparatively limited computing capabilities this unit cannot run complex models, such as those on the Pi 3. Thus, the Raspberry Pi Zero module represents the low-end edge devices in our experiments.

##### Intel Neural Compute Stick 2

The Intel^®^ Neural Compute Stick 2 (Intel^®^ NCS2) unit executes server-grade deep-learning models at the edge level power consumption. It consists of an Intel Movidius Myriad X Vision Processing Unit and 4 GB of RAM. With this accelerator, a Raspberry Pi can run complex models as a GPU or a TPU used in a server environment. Therefore, Raspberry Pi 3 equipped with an Intel^®^ NCS2 represents the high-end edge devices in our experiments [67].

##### Raspberry Pi Camera Module

The Raspberry Pi camera module is intended to capture both still images and high-definition videos. The original Raspberry Pi camera module has an effective resolution of 5 Mega-pixels and supports video recording at 1080@30fps, 720p@60fps and Vga@90fps. Later, in 2016, 8 Megapixel Camera Module v2 was released and currently, the latest version has a high-quality resolution of 12 Mega-pixels. Both early versions supported visible light and infrared versions and however, there is no infrared version for the latest 12-Megapixel model. However, this high-quality camera uses a Hoya CM500 infrared filter and can be removed if needed. The camera module can be connected to Raspberry Pi via Camera Serial Interface (CSI) port and can be accessed via Multi-Media Abstraction Layer (MMAL) and Video4Linux (V4L) APIs and other third-party software such as Picamera Python Library [68].

##### GSM Module Sim 900a

Global System for Mobile Communications (GSM) module sim 900a is a GSM modem that supports Quad-bands GSM850, EGSM900, DCS1800 and PCS1900. The shield sends and receives General Packet Radio Service (GPRS) data through protocols such as TCP/IP and HTTP. It also allows sending SMS, MMS, GPRS and Audio via UART using ATtention (AT) commands [69].

#### 3.1.2. Input Module

The input module consists of two main components, a motion trigger and a camera. Motion trigger detects motions such as movement of a vehicle and activating the rest of the system. The camera captures the images at nighttime without additional visible illumination. The motion trigger uses a passive infrared (PIR) sensor to detect movements. PIR sensor detects the changes in the amount of infrared radiation falling on it and detects the motion. For instance, when a vehicle passes near the sensor, the heat radiation from the vehicle engine fall on the sensor as it enters the sensor’s field of view. When the vehicle leaves the sensors field of view, then it will stop the heat radiation. This causes a change in the amount of infrared radiation falling on the sensor causing the sensor to be activated. A typical PIR sensor can detect a motion, but it cannot recognize the motion. Despite this limitation in many state-of-the-art solutions, PIR sensors are widely used for detection applications such as surveillance systems, automatic lighting, and alarm systems as simple but reliable motion triggers [70,71]. Our design solution uses PIR sensor purely to detect a motion happening near the motion trigger. Whether that motion was caused by a vehicle passing will be recognized by subsequent modules. The sensitivity range of a PIR sensor is normally up to 20 feet (6 m) and therefore, we use a cluster of PIR sensors to widen the sensor range.

To operate the motion trigger, we used an ESP32 micro-controller with integrated WiFi and Bluetooth connectivity, while performing as a complete standalone system with low cost and low power consumption. When a change of the infrared level is detected by the PIR motion sensor, a digital value is passed to the ESP32 module. After recording this value, it sends a signal to the main processing module via Bluetooth as it boosts considerably low power compared to a WiFi connection. However, as Bluetooth is more reliable with short-range devices, the distance between the sensor module and the main module should be kept less than 10 m, while ensuring no obstructions between the two devices.

Until it receives a signal from the motion trigger, the main processing module will be in a standby mode, which helps to reduce the power consumption. After receiving the signal, it goes to the normal operation state. In this state, it uses the camera from the input module to capture images and passes them through the processing module to recognize the license plate. For the camera, we used a Raspberry Pi NoIR camera V2. It is equipped with a Sony IMX219 8-megapixel sensor without an infrared filter. This coupled with an infrared illuminator that captures images at nighttime without using any visible illuminators. Here, the camera sensor is sensitive to not only the visible spectrum but also to the infrared spectrum, without an infrared filter. Thus, it can capture images using the infrared rays reflected by the license plate. However, this camera setup is not as sensitive as purpose build thermal or night-vision cameras thus requiring an infrared illuminator. The main advantage of using this camera setup over such a purpose build camera setup is to produce a low-cost solution.

#### 3.1.3. Main Processing Module

**Figure 3:**
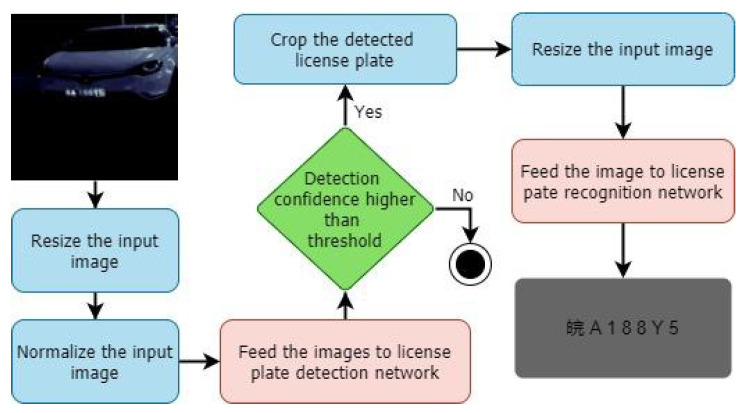
Two-stage license plate recognition pipeline.

The main processing module takes the image from the input module and outputs the license plate content to the communication module. From a software point of view, the main processing module consists of two convolutional neural networks, one that detect and localize the license plate in an image and the second which recognize the content of the license plate. Thus, this is a two-stage license plate recognition process. Figure 3 shows the process flow of the two-stage process of detecting and recognizing a license plate.

The input image is passed through a set of transformations such as resizing and normalizing, before feeding it to the detection model. This model produces two outputs. First, a bounding box description for the license plate and the second, a confidence level value indicating how confident the model is for the bounding box. The confidence value will be high, if there is a license plate in the image, otherwise the value will be closer to 0. If this value is greater than a predetermined threshold value, then the systems moves to the next stage. If not, the image is discarded, and the system moves on to the next image from the camera. If the system discarded all the images within a time period indicating that no vehicle is passed through the system, then the main processing module becomes standby mode waiting to be activated by the motion trigger.

Once an image is passed to the next stage, it is cropped to the license plate bounding box and passed to the recognition model. It will recognize the license plate as a text sequence and passed to the communication module, thus, it can inform the recognized license plate number to the operator, as a text SMS since there is no Internet connectivity in this environment. From a hardware point of view, there are three possible variations for the main processing module, as a single hardware solution may not cover all the possible deployment scenarios. Instead, we propose low, mid and high-tier hardware configurations. Low-tier hardware configuration is intended to be sufficiently inexpensive making large scale mass deployment economical. Higher-tier configuration is more suitable for situations where the unit cost is not that significant and mid-tier is meant to be a middle ground. Computational capabilities increase from low to high tier allowing the use of advanced license plate detection and recognition models giving higher accuracy.

One of the main objectives of this study is to develop an ALPR system for edge devices with minimum cost. Table 3 states the hardware specification for energy-efficient computation and low-latency communication. Each of the configurations uses a common set of hardware including Raspberry Pi camera module V2-8 Megapixel,1080p (USD 23.00), Raspberry Pi power supply (USD 15.00) and GSM module sim 900a (USD 7.00), where the total add up to USD 45.00.

**Figure 4:**
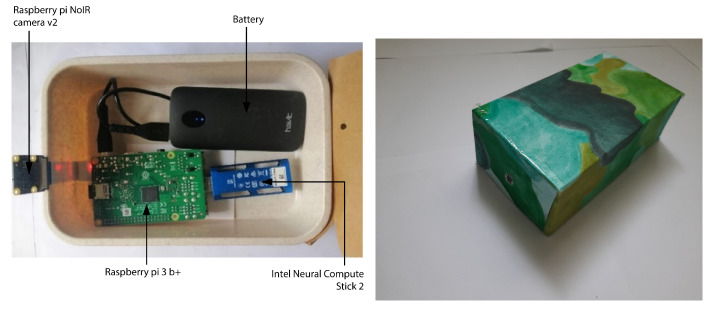
High-tier model (**left**): Internal view, (**right**): Exterior deployment view.

**Figure 5:**
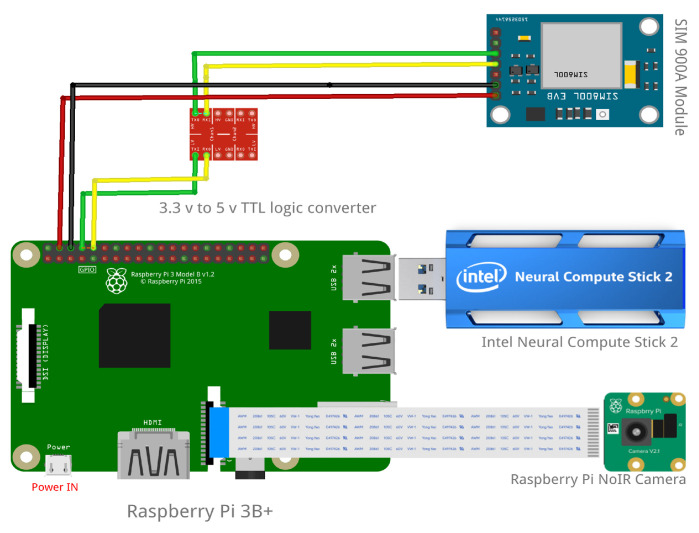
Circuit diagram of the design.

We developed different models for license plate detection and recognition based on neural architecture search strategies as described in Section 3.3, per each tier to exploit the capabilities of different hardware tiers. The appearance and the circuit of the configuration are shown in Figure 4 and Figure 5, respectively.

In the license plate detection process, we developed two models for each hardware tier. One model is optimized for the specific hardware platform using a hardware aware architecture search strategy and another model optimized using a hardware-agnostic architecture search. Both models are small in size, and the required computational power is sufficient to execute on the target hardware, while the hardware-optimized model gives better latency compared to the hardware-agnostic model. However, the hardware-agnostic model can generalize better with other similar hardware setups. In the license plate recognition process, we developed three models based on hardware-agnostic architecture search, representing each hardware tier.

Consequently, the lower-tier configuration uses a Raspberry Pi Zero as its hardware platform. As stated in Section 3.1.1, it is a relatively inexpensive single-board computer with limited processing capabilities. As a result, it is coupled with the simplest detection and recognition models. Mid-tier configuration uses a Raspberry Pi 3 B+ instead of the Raspberry Pi Zero; thus, allows the execution of more complex models and has high computing capabilities giving better accuracy. The higher-tier configuration consists of a Raspberry Pi 3 B+ with an Intel^®^ NCS2. This offloads the execution of convolutions neural networks to the more computationally capable Intel^®^ NCS2 allowing use of computationally expensive but more accurate models.

Figure 4 (left) shows the internal module design of the main processing module along with the camera in the higher-tier configuration and Figure 4 (right) shows the main processing units exterior view, which is designed for a wild environment as explained in the experiment setup with the case study. The exterior view of the main processing module are based on several consideration based on the proposed application domain. The package needs to be compact, thus it can be easily camouflaged and hidden from direct view. At the same time, it must larger enough to store all the components of the system except the motion trigger along with the battery to power them in it. Figure 5 shows the design circuit of the proposed solution.

#### 3.1.4. Communication Module

**Figure 6:**
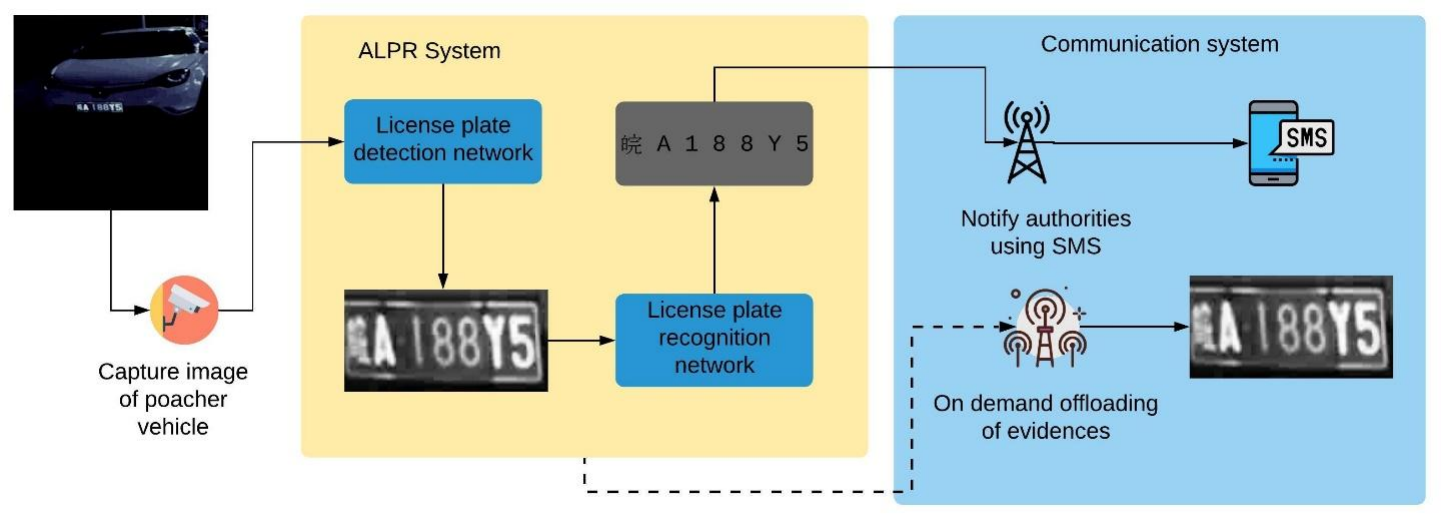
Data flow of the proposed system.

The communication module consists of two main components. The SMS notification system notifies the characters in the recognized license plates to the authorities and the on-demand evidence offloading module offloads images stored within the system. Data flow of these components and the main system is shown in Figure 6. Once a license plate has been successfully recognized by the license plate recognition model, it is passed to the SMS notification system. The SMS notification system uses a sim 900a mini v3.8.2 GSM module connected to the main processing module to send SMS messages. It is connected to the Raspberry Pi’s serial TTL port using the universal asynchronous receiver/transmitter (UART) protocol. Since sim900a is a 5 V device and Raspberry Pi is a 3.3 V device, we used a 5 V to 3.3 V TTL logic shifter to protect the Raspberry Pi.

The on-demand evidence offloading module is designed as a quality-of-life improvement, thus the operators do not require physical connection with the system to offload data. To use this system, the operator sends an SMS message to the system, which enables the WiFi module of the Raspberry Pi. It then searches for a WiFi hot-spot with a predefined Service Set Identifier (SSID) and WiFi Protected Access 2 (WPA2) password. The operator will carry a mobile device that uses the mobile hot-spot functionality to create this hot-spot. After the Raspberry Pi has been successfully connected to the hot-spot operator can access the images stored within the Raspberry Pi in wireless mode and download necessary files. With this system, operators can easily access images stored within the system without a physical connection to the system, which may be difficult due to camouflaged placement of the system.

### 3.2. Environment Simulation Techniques

Generally, a large and diverse dataset supports to train a learning model robustly. This helps to classify data against varying environmental conditions including adverse weather and camera conditions such as location and vibration without the need for fragile explicit image processing steps. There exist such LP datasets such as Chinese City Parking Data set (CCPD) [14] that is used by state-of-the-art models such as Roadside Parking Net (RPNet) [14] and Towards End-to-End Car License Plate Detection and Recognition (TE2E) [64] to achieve different variations. However, these data sets have mainly focused on daytime images. Additionally, curating such a data set for nighttime images is both expensive and time consuming. Therefore, to simulate the night vision, we used a synthetic image generation technique to convert the RGB images of the CCPD dataset to nighttime TIR images. However, we also deployed the working prototype in the actual field to acquire a real nighttime dataset to evaluate the performance of our proposed models.

**Figure 7:**
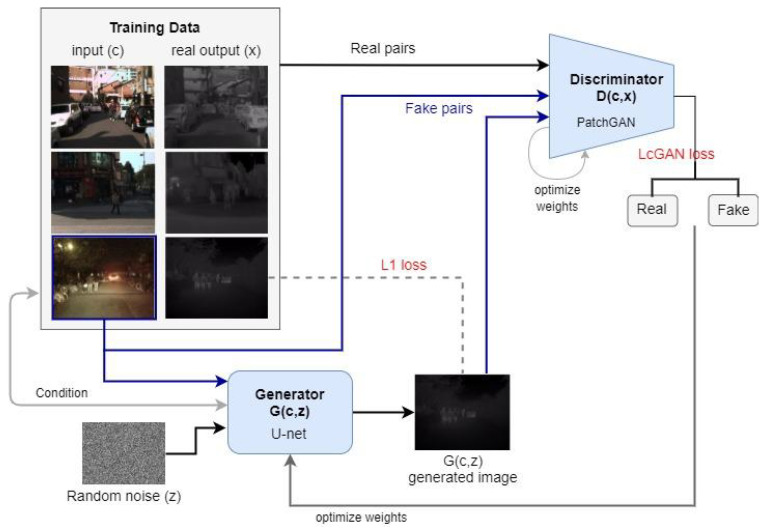
Pix2Pix for nighttime image generation.

The process of generating the synthetic TIR images used by this work follows a method proposed by Zhang et al. [49]. As shown in Figure 7, we used a GAN-based pix2pix model for image translation and provided the model with a paired set of training data that includes matching frames in both RGB and TIR images. To train the model for TIR image translation, we selected the largest available multi-spectral dataset named KAIST [72] that has a significant amount of matching RGB and TIR images. Finally, we trained the pix2pix model and initialized the weights from a Gaussian distribution with a mean 0 and standard deviation of 0.02. The input images were enlarged to 480 × 480 pixels and the network is trained for 100 epochs with a decaying learning rate of 0.0002, lambda_l1 of 120.0 and keeping other parameters the same as the original pix2pix study. Then we used this trained pix2pix model to translate the daytime RGB images of the CCPD dataset [14] to TIR and used that synthetically generated nighttime images of CCPD to train the detection models of our pipeline. To train the recognition models, we required comparatively high-quality nighttime images of the license plates. Therefore, we converted the RGB images to grey-scale using matplotlib Python library and set the colour map to grey. Herewith, we preserved the image quality and avoided generating incomplete license plate characters that are impossible to read.

### 3.3. License Plate Detection and Recognition Algorithms

In this paper, we use two differential neural architecture search (DNAS) strategies to automate the architecture modelling for detection and recognition neural networks. We define the neural architecture search problem as a bi-level optimization problem as in Equation (1),

(1) $$\min_{a\in A}\min_{w_{a}}L\left(a,w_{a}\right)$$

where *A* is the set of possible neural network architectures referred to as the architecture space and $w_{a}$ is the set of weights for the selected architecture *a*. Loss function *L* takes into account both the resource use and model accuracy. In this work, we consider three main factors related to neural architecture search, namely search space, search strategy, and performance estimation strategy.

#### 3.3.1. Search Space

The proposed neural architecture search (NAS) process uses a coarser search space with “neural blocks” selected on the existing understanding of the domains such as license plate recognition and object detection. Therefore, we selected 4 types of neural blocks: (1) RPNet blocks [14], (2) MobileNet blocks [73], (3) Inception blocks, and (4) Identity connections. The RPNet blocks were considered to be they currently serve the state-of-the-art results in the automatic license plate recognition domain. The selection of MobileNet blocks was based on two major factors. First, it is one of the backbone architectures used in most of the object detection problems and secondly, it is lightweight and runs efficiently in resource-constrained environments such as mobile devices or other devices with low computational power and memory space. The Inception models are uniform, simplified and heavily engineered architectures that introduce the concepts for “wider” networks instead of “deeper”. One can consider the search space as the set of possible permutations of these blocks that can run on the edge device. Although selecting a “finer” search space may have resulted in better performance, we decided against it because that will lead to a much larger search space requiring more computational time to perform the architecture search.

#### 3.3.2. Search Strategy

In this study, two neural architecture search strategies namely PC-DARTS (Partially connected—Differentiable architecture search) [13] and FB-Net (Facebook-Berkeley-Nets) [12] are explored to discover the neural network architectures for the license plate detection and recognition modules optimized for memory-constrained embedded devices. Our previous work has presented the detailed implementation aspects of the LP-net architecture used for this study [57].

We used PC-DARTS as a hardware-agnostic neural architecture search strategy. Thus, it optimizes the architecture considering only the input and the target output, independent of the hardware platform. We introduced a hard upper limit to the memory use in *A* based on the target device. This ensure all possible values of *a* can be run on then given target. Rational for performing architecture search in a hardware-agnostic manner is to develop models that will perform well on targets similar to the intended target by preventing overspecialization to the intended target. PC-DARTS defines its stochastic super-network as a directed graph where vertices represent tensors and edges represent operation in the search space.

**Figure 8:**
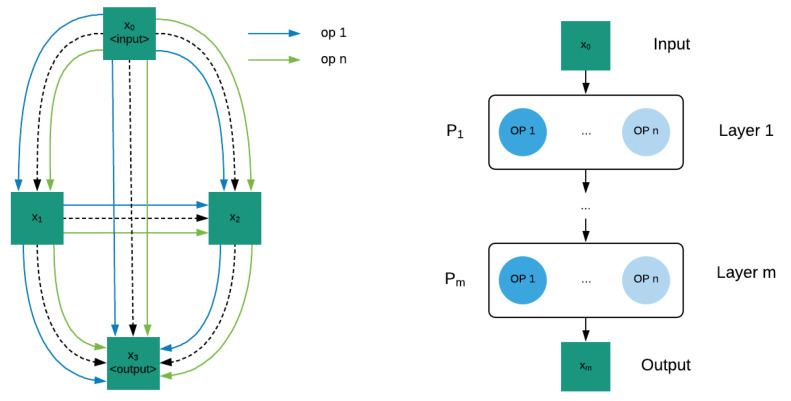
Stochastic super-network (**left**): PC-DARTS, (**right**): FB-Net.

Figure 8 (left) shows a simple case with only 2 intermediate tensors namely $x_{1}$ and $x_{2}$. The tensor $x_{0}$ is the input to the super-network and tensor $x_{3}$ is the output of the super-network. We call the number of intermediate tensors as the depth of the network in our implementation. As shown in the figure each tensor is connected to every one of its predecessors using all the operations in the search space. For brevity we have shown only op 1 and op n in the figure. For our architecture search process these operations are the neural blocks described in the previous section.

(2) $$x_{j}=\sum_{i<j}\sum_{o\in O}\alpha_{\left(i,j,o\right)}o\left(x_{i}\right)$$

Value of each tensor $x_{i}$ can be defined using its predecessors as shown in Equation (2). Here we are using value of subscripts to represent the order of tensors and *O* represents the set of operations in the search space. We call the value of $\alpha_{(}i,j,o)$ as the architecture weight of operation *o* for edge (i,j). These weights represent the probability of connected each tensor with its predecessor *j* using operation *o*. Therefore, we used a SoftMax distribution to represent these weights. We call the set of all such architecture weights as the architecture weights of the super-network ($w_{\alpha }$). Each individual operation such a convolution can have their own weights and the set of all such weights in the super-network is known as the operation weights of the super-network ($w_{\theta }$). We can then find the optimal values for $w_{\alpha }$ and $w_{\theta }$ using bi-level optimization as given in Algorithm 1. Once this optimization has converged, we can find the optimal architecture by performing argmax on architecture weights.
**Algorithm 1**: Bi-level optimization.
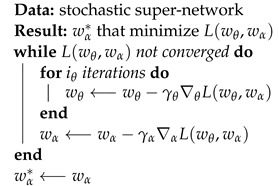

$w_{\theta }$: operation weights
$w_{\alpha }$: architecture weights
$\gamma_{\theta }$: learning rate for operation weight update
$\gamma_{\alpha }$: learning rate for architecture weight update
$L(w_{\theta },w_{\alpha })$: loss
$i_{\theta }$: number of iterations for inner optimization

The FB-Net was used as the hardware sensitive search strategy that produces optimized models for a specific hardware platform. Hence, FB-Net-based models use special hardware characteristics of the target platforms to reduce their latency. However, the performance can be reduced, if these models are used on a different hardware platform other than the platform considered for the optimization due to overspecialization for the intended target. As a result, models developed using FB-Net gives us better hardware use at the cost of generalizability across different hardware platforms.

Similar to PC-DARTS FB-Net also represent the search space as a stochastic super-network. However, it is more similar to a typical feed forward network as shown in Figure 8 (right). Each layer takes the output of the previous layer $x_{i-1}$ and apply operation as shown in Equation (3) to obtain its output $x_{i}$. *O* is the set of all operations in the search space.

(3) $$x_{i}=\sum_{o\in O}\alpha_{\left(i,o\right)}o\left(x_{i-1}\right)$$

We call the value $\alpha_{\left(i,o\right)}$ architecture weight of layer *i* with respect to operation *o*. Set of all such weights is given by $p_{i}$ as shown in Equation (4). We define the set of all such $p_{i}$ values as the architecture weights of the stochastic super-network($w_{\alpha }$). We then used the previously given bi-level optimization to obtain the optimal architecture similar to PC-DARTS.

(4) $$p_{i}=\alpha_{\left(i,1\right)}\forall o\in O$$

#### 3.3.3. Lite LP-Net Architectures

**Figure 9:**
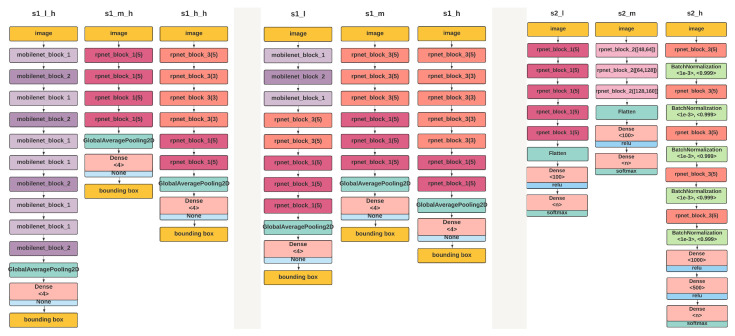
Model Architectures (**left**): hardware-optimized detection, (**middle**): hardware-agnostic detection, (**right**): recognition subnetworks.

We have designed and developed a set of optimal learning models that can be deployed in edge devices with low processing power and worked without Internet connectivity. The proposed Lite LP-Net family of models consists of (1) hardware-optimized LP detection model, (2) hardware-agnostic LP detection and (3) LP recognition subnetworks as shown in Figure 9. The naming convention of the models is detailed in Table 4 and Table 5. We used the tensorflow.keras.layers API and the default parameters as in TensorFlow version 2.3.0. Moreover, as stated in Section 3.1.2, the hardware-optimized LP detection model is implemented following using FB-Net (Facebook-Berkeley-Nets) [12] algorithm and the other two models were based on PC-DARTS [13]. These were implemented for three hardware configurations namely low, mid, and high tier, as described in Section 3.1.1.

In addition, we have used a novel differentiable neural architecture search (NAS) process based on PC-DARTS and FB-Net to develop the models. The advantage of using differentiable architecture search over commonly used methods such as reinforcement learning, and evolutionary algorithms is a significant reduction in GPU hours required to search of neural networks. To the best of our knowledge, this is the first time such techniques have been used for the development of models for license plate recognition in edge devices such as Raspberry Pi and neural compute stick, which has different computational capabilities and requires different model designs and optimizations.

The LP detection models are designed to predict the bounding boxes of the license plate image. As shown in Figure 9 (left) and (middle), the detection model uses 6 different models. The hardware-optimized and hardware-agnostic models are designed to reduce the latency and increase the accuracy, respectively. Considering the application domain considered for this study, we recommend the model that supports low latency. These hardware-optimized models are implemented by applying NAS with the FB-Net algorithm as described in Section 3.3. The hardware-optimized model for each tier is selected based on the latency values calculated for each hardware configuration and applying NAS. Although hardware-optimized models provide low latency in the processing, these models can give a subpar performance in similar but not identical processing units. Thus, hardware-agnostic models were designed to handle this variability. The implementation of these models is based on the PC-DARTS algorithm and optimized to increase detection accuracy without regard to processing latency.

The LP recognition models provide a sequence representing the content, given a cropped image of the license plate. As shown in Figure 9 (right), this study presents three 3 hardware-agnostic models for LP recognition, by following the same process as used for the hardware-agnostic detection models. We applied two design paradigms. (1) The model based on the Tuple-based End-to-end (TE2E) [64], uses a single model to predict all the characters in the image. Since it shares parameters when recognizing each character, the memory consumption is low. (2) The model based on Roadside Parking Net (RPNet) [14], uses a separate subnetwork for each character in the license plate. Since the separate subnetworks cannot share the parameters, the memory consumption is high. The optimal architectures were obtained by training the stochastic super-networks as described in Section 3.3.2. The entire set of characters in the license plate is the input for each subnetwork and the consecutive output values of each subnetwork form the recognized license plate number. The subnetwork-based approach has outperformed the single model approach, based on the experiments done for each hardware configuration.

#### 3.3.4. Performance Estimation Strategy

The performance estimation strategy is used to identify the optimal architecture among the selected architectures. Generally, the evaluation strategy of NAS has a bi-level optimization problem as in Equation (1). Thus, for a given input the aim is to learn an optimal architecture *a* to obtain a given output, and the associated weights *w* within all the mixed operations. In our experiment, the input to the NAS is either an image directly from the camera or an image of the cropped license plate, and the output is either the bounding box of the license plate or the sequence representing characters in the license plate. However, unlike in PC-DARTS that considers the accuracy of a given architecture only, the loss function used in FB-Net is more thorough and reflects both accuracy and latency of an architecture on a target hardware. Thus, the architectures searched using FB-Net algorithm become hardware sensitive. In this study, we used the same latency aware loss function as in the original FB-Net implementation.

First, a latency table is created for the execution of each operation on the target hardware. Then, we use the latency lookup table and calculate the latency of layer *i* using value $p_{i}$ as shown in Equation (5).

(5) $$LAT\left(p_{i}\right)=\sum_{o\in O}lat_{o}\alpha_{\left(i,o\right)}$$

In Equation (5), $lat_{o}$ refers to the latency of operation *o* read from the latency lookup table. Then we obtain the latency of the super-network, $LAT(w_{\alpha })$, by summing up the latency values for all the layers in the network. Therefore, we include this latency term in the bi-level optimization algorithm to obtain a hardware sensitive architecture search process.

## 4. System Evaluation

### 4.1. Data Set

Two experiments have been done to test the performance of the proposed detection and recognition system using two different data sets: a simulated and a real nighttime data set as listed in Table 4. The first experiment was done on a Chinese City Parking Dataset (CCPD) that has over 200,000 images collected from a roadside parking from 07.30 a.m. to 10:00 p.m. covering different illumination and environmental conditions during the day. However, still a large portion of the CCPD dataset is also taken under daylight similar to most other datasets available for LP detection. Therefore, due to the scarcity of a publicly available nighttime LP dataset and curating such a large nighttime dataset is both expensive and time consuming, we created a synthetic nighttime dataset using the CCPD daytime images as comprehended in Section 3.2. Although CCPD which is the largest LP image dataset has complex background conditions when compared to an LP image captured in a wild environment, training with this dataset is beneficial to obtain a well-trained model for LP detection, as the actual image is less complex than the trained dataset. With the synthetically generated CCPD data set, we used a five-fold cross validation, where each fold consists of 40,000 images.

The second experiment was done using a real-world Sri Lankan data set which was collected specifically for this considered use case of wild environment conditions. The created real-world nighttime data set contains 100 images and was collected between 8 p.m. to 4 a.m. Then we used this collected data set to perform transfer learning on our models to train them for Sri Lankan license plates and then validated the performance of them against local license plate numbers. However, as the main focus of this study was to build an ALPR system to work with resource-constrained environments, the created dataset does not include any complex weather conditions.

### 4.2. Experiment Setup

A simulation of a poacher vehicle detection case study is used to evaluate the effectiveness of the proposed approach. This experiment has been performed using the CCPD dataset with 200,000 daytime license plate images [14], and the corresponding synthetically generated nighttime license plate images following the process described in Section 3.2. In addition, the proposed model is practically tested in a real nighttime environment with 120 vehicle images. The hardware configuration specifications have described in Section 3. The software configuration consists of Raspberry Pi OS (32-bit) version August 2020, TensorFlow lite version 2.1.0 and Python 3.7.3. We used Open-VINO version 2019.3.376 to convert TensorFlow models that were compiled using TensorFlow version 2.2 into intermediate representations for the Intel^®^ NCS2. The model training and evaluation codes for Lite LP-Net is available in GitHub repository [74].

### 4.3. Model Performance

The performance of deep-learning models used for license plate recognition was measured under two broad categories. We measured the model correctness using the three datasets and efficiency while achieving the task it was designed to perform. For stage one (detection) models, we used an average precision at a fixed Intersection over Union (IOU) threshold, a metric typically used for object detection as the evaluation metric. Also, to measure the correctness of the stage 2 (recognition) models, we used a more relaxed metric of accuracy, and we considered a prediction to be accurate if and only if every single character in the license plate is recognized correctly. Model efficiency is measured using two parameters, model size and model latency. The model size is measured considering the size of the TensorFlow Flatbuffer that estimates the required RAM to execute the model. Model latency is calculated by considering the average time takes to process a single image. This can also be viewed as a proxy to the computational complexity of the model.

Results of these experiments are shown in Table 5 and Table 6 for the detection and recognition stages, respectively. The model names ending with h, m and l represent high-tier, mid-tier and low-tier configurations, respectively. Each hardware tier in the detection process contains two types of models namely hardware-optimized using FB-Net [12] and hardware-agnostic using PC-DARTS [13].

**Figure 10:**
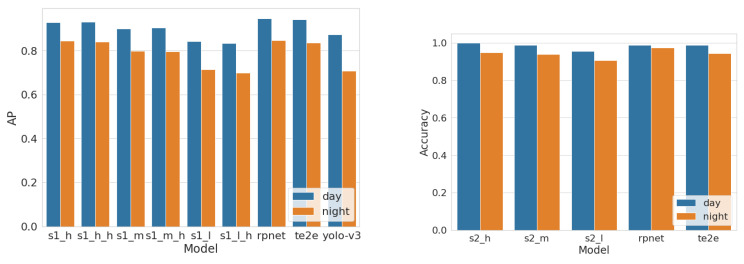
Model accuracy on the synthetically generated dataset (**left**): detection, (**right**) recognition.

First we evaluated the day and nighttime performance of each model using the original CCPD [14] data set and the synthetically generated nighttime data set. Figure 10 compares the detection and recognition models’ performance for day and nighttime data. According to the reported values, all the models have shown high accuracy in both day and night conditions, while high-tier models have shown better accuracy than the other models. Additionally, we have tested our models against some state-of-the art ALPR systems such as RPNet [14], TE2E [64] and a general object detection models such as yolo-v3 [46] for a better comparison. We can also observe that the proposed detection models, especially higher-tier models show performance close to the current state-of-the-art server-grade models such as RPNet, although our models are designed specifically for low resources. At the same time, all models except the lower-tier ones show superior performance to Yolo-V3 [46], which is a popular general-purpose object detector that has been used in several license plate detection studies [44,45]. Meanwhile, the same trends can be observed for the recognition models as well. Higher-tier models perform better than lower-tier models and unlike with detection higher-tier models actually outperform the current state-of-the-art models such as RPNet.

In the detection stage, the hardware-optimized models have lesser latency than their corresponding hardware-agnostic models (s1_h, s1_m and s1_l). Overall, the low model sizes shown the ability to execute these models in edge devices with low resources.

**Figure 11:**
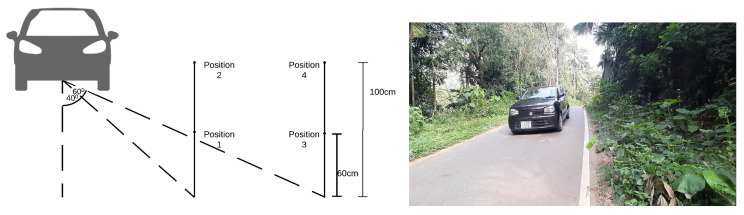
Camera positions (**left**) and sample deployed image (**right**).

Moreover, we measured the model robustness against variations of camera position to identify the impact of the camera angle and elevation on the performance of the system. This experiment aims to validate that the model performance does not change significantly with the changes in the camera position. Metrics related to model efficiency are functions of the model and the hardware solution, thus independent of the camera position. In contrast, we check whether the model correctness metrics are affected by the camera position. To validate the impact of the camera position on the model accuracy, an experiment was carried out by driving a vehicle at a speed in the range of 20–30 km/h towards the camera. The camera was positioned in one of the four positions as shown in Figure 11 (left). Angle measurement indication is between the centre of the license plate and the camera when the vehicle is 20 m away from the camera. We started the test when the vehicle is 20 m away from the camera and executed the test until the vehicle left the view range of the camera. During this time, we sampled the video stream at the rate of 10 frames per second and identified the number of correctly recognized license plate numbers.

The considered environment is a rural area with many trees and bushes, thus can be simulated as a wildlife sanctuary. Figure 11 (right) shows a sample image taken under the same conditions from camera position 1 during daytime to better illustrate the environmental conditions under which this experiment was performed. The actual images used for the accuracy results are taken at the same location during nighttime (8 p.m.–10 p.m.) on a moonless night (13 January 2021).

In this experiment, we used a Raspberry Pi NoIR camera for capturing nighttime images. The functions of the Pi NoIR camera are same as a regular camera; however, it does not employ an infrared filter for IR-Blocking, therefore allowing it to use in infrared photography in general. However, one of the main benefits of using a NoIR camera is its ability to be used in both daytime and in complete darkness as well. Moreover, it is also relatively less expensive compared to a regular IR camera module, where one of the main focus of this study is a low-cost solution. Though a NoIR camera can see better in a low light environment even without the assistance of an IR illuminator, using an infrared light source (illuminator) that is completely invisible to the human eye, can ensure a clearer image in the total darkness. Therefore, in this design, we have used an infrared illuminator that is invisible to the naked eye for better performance. Thus, our solution gives the system the most challenging conditions because there are no visible illumination sources.

**Figure 12:**
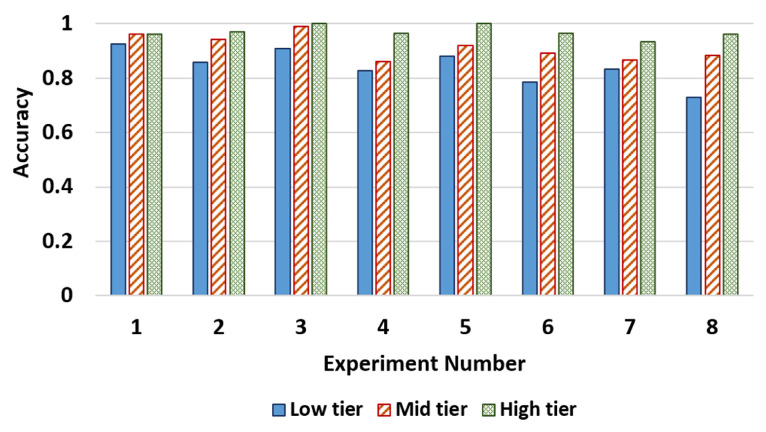
Model accuracies of each experiment.

Results of this experiment are shown in Table 7. The proposed model performance is not affected adversely depending on the camera position. Furthermore, as shown in Figure 12, the higher-tier models have shown better accuracy. As we can see from this experiment, the proposed model is robust against variations of camera elevation and angles giving results that are similar to each other irrespective of camera position. This is to be expected, because the CCPD [14] dataset contains images taken from handheld devices giving high variation in terms of both elevation and camera angle.

### 4.4. Hardware Performance

Since the proposed solution is supposed to be a battery-powered system that will be deployed in a wild environment, the metrics battery life and power consumption are used to evaluate the hardware performance of the edge devices. We measured the peak power consumption where the processing unit executes at maximum load, using the input power via the USB interface to Raspberry Pi devices. Since the camera and Intel^®^ NCS2 (where applicable) is powered via the Raspberry Pi, this gives us the power requirement for a minimum ALPR system with both input and processing capabilities. The worst-case power consumption over a general case is considered due to the following reasons:

1. The probabilistic estimation of the number of vehicles passing through an operation unit is not readily available for a given case study. Thus, we considered the maximum possible processing load on the unit for a general case.
2. The worst-case power consumption gives an upper bound for the unit’s power consumption. Thus, using a power supply that satisfies the maximum power requirements can satisfy the power consumption of the unit under any other condition.

This measure includes the power consumption of all the processing units required to execute the model including its input devices. As shown in Table 8, there is an increase in the power consumption, when moving from the Raspberry Pi Zero (low-tier) to Raspberry Pi 3b+. Although we observed an increase in peak power consumption when the Raspberry Pi 3b+ was combined with the Intel^®^ NCS2, it was a relatively smaller increase.

To measure the expected battery life of a typical deployment, we used a 10,400 mAh battery to power all the components of the system. We charged the battery to 100% and executed the system continuously until it runs off the power. We measured the time taken to drain the battery completely using the timestamp of the last image recorded by the system. For each hardware tier, we repeated this experiment for a week and measured the average battery life as shown in Table 8. The lower-tier hardware has significantly better battery life compared to mid and higher-tier configurations. The most interesting observation in this experiment was that the higher-tier system has a better battery life compared with the mid-tier unit even though it had a higher peak power consumption. A possible reason for this could be the better computing performance of the higher-tier model with the Intel^®^ NCS2. Hence, higher-tier models do not reach their peak load as often as the mid-tier models that operate closer to maximum load with the Raspberry Pi, thus higher-tier models consume less energy. With the knowledge of the power consumption and battery life of the models, a suitable battery that meets the deployment requirements such as cost, external dimensions, battery recharge and replacement frequency can be selected in practice. Although 13 h of battery life seems low in the high tier, the recorded time is the sustained use time, where the system is taking pictures and processing them in a continuous manner. However, in a forest environment, where there will not be many vehicles passing by, we have installed a motion trigger to keep the device in a standby mode when no vehicle is detected for a fixed amount of time. Therefore, the actual battery life is much longer than this use time. Additionally, the system design can be even modified to use solar recharging batteries.

Furthermore, we evaluated the communication systems of the proof-of-concept hardware solution. We deployed the proposed models under operational conditions and test the correctness of sending SMS messages and the data offloading module. Thus, we have verified that the purposed hardware solution meets the requirements of the case study.

## 5. Discussion and Lessons Learned

### 5.1. Study Contributions

We presented an innovative approach to detect and recognize license plates automatically for embedded platforms with limited computational and memory capacities. The overall aim of this study is mainly twofold: (1) develop models for license plate detection and recognition that gives competitive results to the server-grade hardware solutions, while still being efficient enough to run on low-resourced, low-cost embedded platforms and (2) develop a system that is energy efficient and viable to be deployed in wild or remote areas without reliable Internet connectivity or direct power supply. The proposed approach has achieved the following objectives;

- Designed and developed a lightweight and low-cost night-vision vehicle number plate detection and recognition model with competitive accuracies.
- Developed a license plate reading system capable of operating without Internet connection and powered by batteries for an extended period. Thus, supported mobile communication with minimum resources.
- Supported SMS sending that contains the identified license plate number to a given phone number (e.g., send to the wildlife department in the considered case study).
- Designed in small size in appearance and deployed discreetly in the field. Thus, in the considered case study, the poachers may not notice these camera traps and equipment.
- Analysed the trade-offs and explored the impact of the constraints such as accuracy and power consumption.
- Maximized resource use and minimized the end-to-end delay.

We have shown the use of a novel family of neural networks called the Lite LP-Net model for both licenses plate detection and recognition, which are light-weighted and optimized for edge devices. As another novel contribution, we used an infrared blaster to capture nighttime images in the dark. It captures the license plate using its illumination, without visual illumination at nighttime. We have also presented a case-study-based approach as a proof of concept for the use of proposed models in real-time applications in the wild. The experiment results have shown the system’s robustness to variations in the angle and its high recognition accuracy at nighttime. Providing a basis for future research on nighttime license plate recognition, this study has also presented a synthetic data generation technique to create a versatile nighttime license plate dataset with publicly available RGB images of license plates. The main advantage of this approach is that it helps to mitigate the problem with the scarcity of large and diverse nighttime LP datasets.

Moreover, as shown in Figure 11 (left), the system design has considered the technical aspects such as angle of the camera, distance to the camera, camera location. The models detect and recognize the license plate in constrained environments with different vehicle speeds and lighting conditions. Thus, the model can execute on edge devices with low-resource requirements and showed competitive accuracy values compared to server-grade related systems. However, the proposed solution can be further extended to train learning models for different image variations with constraints environments such as diverse weather conditions, and complex parameters such as license plates rotations to develop robust models. Furthermore, these energy-efficient and low-latency communication and computation models can be deployed at a low cost, such that the total cost of low-tier and high-tier models are USD 63 and USD 146, respectively.

Based on the considered case study, model size is a main limiting factor when deploying the license plate recognition models in edge devices, and higher latency may be tolerable. To execute the inference, the model size should be smaller than the device memory. As shown in Table 5 and Table 6, our proposed model sizes are significantly smaller, hence can execute in memory-constrained edge devices. Moreover, although, the higher-tier models have high power consumption, they execute more accurate models and have smaller latency compared to the lower-tier hardware configurations. We have simulated an experiment for the case study of the poacher vehicle detection system. Such a system might support the wildlife in minimizing the rate of losing their existence and violent matters. It will, directly and indirectly, affect the rights of the wildlife by assuring the security of the wild animal’s lives. Thus, reduces damage done to wildlife in reserves by making prosecution of poachers easier. Accordingly, this approach can be used to identify vehicles number plates in remote locations without access to the Internet and power grid. A similar system can be used for any scenario that requires reading license plates such as parking lot management, traffic management.

### 5.2. Solution Assessment

The problems of automated license plate recognition have many proposed solutions. However, it cannot be denied that most of these prevailing solutions are limited to unconstrained environments with higher computational capabilities and memory capacities. Despite their accuracy and latency in server-grade hardware, most of the state-of-the-art solutions in the ALPR domain are not implementable on the embedded platforms due to their memory and energy requirements. For instance, RPNet [14] model currently serves the state-of-the-art results in the ALPR domain but still, it is tested on PCs with eight 3.40 GHz Intel Core i7-6700 CPU, 24 GB RAM, and one Quadro P4000 GPU. Thus, though it achieves over 90% accuracy for plate recognition, it cannot be executed on a low-cost edge platform such as a Raspberry Pi. However, in this study, we have proposed a system that is implementable on these embedded platforms but still showing competitive results to the server-grade solutions.

Since the solutions built on the server-grade hardware requires more memory requirements and computational power, the researchers are encouraged to build lightweight ALPR systems to execute these solutions on edge devices for practical scenarios. To assess the significance of our approach, we have compared the proposed solution with the existing embedded ALPR systems as given in Table 9. Since most studies do not report energy consumption or memory requirements for their methods, a direct comparison for these values was not possible. However, our solution has shown competitive performance and the subsequent studies may use our values as a reference to guide future research. In contrast to existing studies, the proposed solution is not limited to one specific edge platform. Thus, our approach is generalized over three hardware tiers and any edge device within the specifications or the computational capabilities of either of these tiers can effectively use the proposed models.

Moreover, as shown in Table 5 and Table 6, the proposed higher-tier detection models show performance close to the current state-of-the-art, RPNet [14]. At the same time, all models except the lower-tier ones show superior performance to Yolo-V3 [46], which is a popular general-purpose object detector that has been used in several LP detection solution designed to execute on server-grade hardware [31,65]. Similarly, considering the recognition models, the higher-tier models perform better than the lower-tier models. In contrast to the detection stage, these higher-tier models outperform the current state-of-the-art models such as RPNet [14]. Here, both RPNet [14] and TE2E [64] are single-stage models that are designed to both detect and recognize LP with a single forward pass. This shows that our models are competitive with the existing state-of-the-art solutions in terms of accuracy which was the research objective.

Furthermore, our solution is tested for both daytime and nighttime performance, while most of the other methods are limited to daytime performance only. We have also proved the real-world usability of our system in the wild by holding a case study and has shown the system’s robustness to the variations in the camera angle and different illumination conditions. The model performance can be analysed further using a confusion matrix, as it shows a summary of the number of correct and incorrect predictions with count values for each class. Additionally, we validated our solution with a large and diverse dataset with over 200,000 images in different conditions. Moreover, we have obtained lesser execution time when compared to other embedded systems, thus showing that our solution is more suitable for real-time applications. Furthermore, we have managed to maintain the peak power consumption of the high-tier solution to 6.2 W and the average battery sustained use time to 13.04 h even in the worst-case. In addition, the low-tier solution with 0.8 W power consumption has shown a battery use time of 132.15 h. Thus, the proposed ALPR solution is lightweight, energy efficient, low cost and works in real time.

However, the study has not been tested in different weather conditions and noisy environments as the main focus of this study was to design and develop an ALPR model to be deployed in low-resource settings. Additionally, this study has provided a solution to be deployed in the wild, where there is no stable Internet connectivity or a direct power grid, thus leaving SMS as the only possible communication method. Therefore, although the cloud providers such as Amazon Web Services (AWS) provide edge computing services for specific edge use cases such as this, still, they do not support the resource-constrained environments as considered in this study.

## 6. Conclusions

This paper presents the realization of an automatic license plate recognition system implemented on embedded devices with limited resources. We exploited hardware-agnostic and hardware-efficient neural architecture search strategies to discover a novel set of neural networks for license plate detection and recognition that are efficient enough to execute on edge platforms. Overall, the proposed system has shown robustness to variations in angle, extreme illumination changes such as day and nighttime, and achieved competitive results to the state-of-the-art server-grade hardware solutions. Therefore, our results are significant while considering the restrictions of an embedded system. Additionally, the proposed system is suitable to be deployed in a wild environment, since it does not rely on the Internet connection for communication or a direct power grid for operation. Moreover, we created a synthetic nighttime license plate data set with a widely used Chinese City Parking Data set (CCPD) and a small-scale real nighttime dataset for Sri Lankan license plates that reflects real-life conditions. Additionally, for a fair comparison with the existing server-grade hardware solutions designed for daytime performance, we have evaluated our system against a large daytime dataset. Furthermore, for the generalisability of the solutions over different hardware configurations, we proposed models for three hardware configurations as low, mid and high considering their computational capabilities and the cost.

This study can be extended to customize the neural architecture search process for different hardware platforms. With a one-shot model architecture search strategy such as SMASH [75], the search time for discovering models optimized for any hardware platform can be reduced to O(1) time. Regarding the accuracy of the detection and recognition processes, even though our results are considered reliable, it would be compelling to evaluate the system on different LP datasets for further refinement. Furthermore, the proposed system can also be extended for applications such as illegal license plate identification by compared to an external data source, which would be a promising direction to further explore.

## Figures and Tables

**Table 1:** Summary of the related LP recognition studies on edge platforms.

Related Study	Description	Techniques	Type (D/N/S)	Performance
[15]	Use a NVIDIA Jetson TX1 embedded board with GPU. Provides LP recognition without a detection line. Not robust to broken or reflective plates.	AlexNet (CNN)	D	AC = 95.25%
[16]	Real-time LP recognition on an embedded DSP platform, Operation under daytime condition with sufficient daylight or artificial light from street lamps, High performance with low image resolution.	SVM	D	F = 86%
[17]	Real-time LP recognition on GPU powered mobile platform by simplifying a trained neural network developed for desktop/ server environment.	CNN	D, N, S	AC = 94%
[18]	Implemented in a Raspberry Pi3 with a Pi NoIR v2 camera module. Robust to angle, lighting and noise variations, Free from character segmentation to reduce errors in character mis-segmentation.	CNN	D, S	AC = 97%
[19]	A portable ALPR model trained on a desktop computer and exported to an Android mobile device.	CNN	D	AC = 77.2%

**Table 2:** Comparison of studies with synthetic and nighttime images.

Study	NT	Syn.	Synthesised Method	Performance
[20]		✓	GAN-based	AC = 84.57%
[21]		✓	GAN-based	AC = 91.5%
[22]		✓	Augmentation (rotation, size and noise)	AC = 62.47%
[23]		✓	Augmentation, superimposition, GAN-based	AP = 99.32%
[24]		✓	Illumination and pose conditions	R = 93%
[25]		✓	Random modifications (colour, blur, noise)	AC = 99.98%
[26]	✓	✓	Random modifications (colour, depth)	AC = 85.3%
[27]	✓	✓	Intensity changes	FN = 1.5%
[17]	✓	✓	Illumination and pose conditions	AC = 94%
[28]	✓			AC = 96%
[29]	✓			AC = 93%
[14]	✓			AP = 95.5%
[30]	✓			F = 98.32%
[31]	✓			AC = 95.7%
[32]	✓			AC = 93.99%
[33]	✓			AC = 92.6%
[34]	✓			AC = 86%
[35]	✓			AC = 96.2%

**Table 3:** Hardware tier details.

Hardware Tier	Specification	Cost (as of January-2022)
Low-tier	Raspberry Pi Zero	USD 10.60
Mid-tier	Raspberry Pi 3 B+	USD 38.63
High-tier	Raspberry Pi 3b+, Intel Neural Compute Stick 2	USD 38.63 + USD 89.00

**Table 4:** Detailed summary of the data set.

Data Set	CCPD Day Time Image	Synthesised Nighttime Image from CCPD	Sri Lankan LP Images-Day Time	Sri Lankan LP Images-Nighttime
Sample image	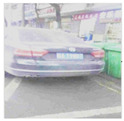	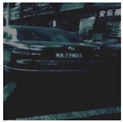	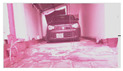	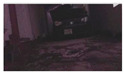
No. of images	200,000	200,000	100	100

**Table 5:** Performance results of the detection model.

Model	Resource	Performance Measure				
Name	Requirement	Latency (s)	Model Size (MB)	AP (Daytime)	AP (Synthetic)	AP (Real)
s1_h	Raspberry Pi 3b+, Intel^®^ NCS2	0.012	0.7776	0.9284	0.8451	0.85
s1_h_h	Raspberry Pi 3b+, Intel^®^ NCS2	0.011	0.8707	0.9299	0.8401	0.9
s1_m	Raspberry Pi 3b+	0.157	0.6869	0.9005	0.7982	0.85
s1_m_h	Raspberry Pi 3b+	0.004	0.6830	0.9029	0.7962	1.0
s1_l	Raspberry Pi Zero	4.54	0.5568	0.8422	0.7146	0.95
s1_l_h	Raspberry Pi Zero	4.08	0.5625	0.8327	0.6987	0.95

**Table 6:** Performance results of the recognition model.

Model	Resource	Performance Measure				
Name	Requirement	Latency (s)	Model Size (MB)	Accuracy (Daytime)	Accuracy (Synthetic)	Accuracy (Real)
s2_h	Raspberry Pi 3b+, Intel^®^ NCS2	0.021	4.5	0.9987	0.9476	0.9873
s2_m	Raspberry Pi 3b+	0.148	11.7	0.9877	0.9382	0.9882
s2_l	Raspberry Pi Zero	6.2	4.5	0.9565	0.9054	0.9586

**Table 7:** Model performance with respect to the camera position (Number of correctly identified images).

Experiment	Number of Images	Number of Correct Images			Camera Position
		Low-Tier	Mid-Tier	High-Tier	
1	27	25	26	26	1
2	35	30	31	34	1
3	33	30	31	33	2
4	29	24	25	28	2
5	25	21	23	25	3
6	28	22	25	27	3
7	30	25	26	28	4
8	26	19	23	25	4

**Table 8:** Hardware performance of each configuration.

Hardware Tier	Power Consumption (W)	Average Battery Life (h)
Low-tier	0.8	132.15
Mid-tier	5.15	11.03
High-tier	6.2	13.04

**Table 9:** Comparison with the related studies.

Study	Dataset	Resource Requirement	Accuracy	Latency
Lee et al. [15]	Nearly 500 images	NVIDIA Jetson TX1 embedded board	95.24% (daytime)	N/A
Arth et al. [16]	Test set 1: 260 images Test set 2: 2600 images Different weather and illumination types	Single Texas Instruments TM C64 fixed point DSP with 1MB of cache, Extra 16 MB SDRAM	96% (daytime)	0.05211 s
Rezvi et al. [17]	Italian rear LP with 788 crops	Quadro K2200, Jetson TX1 embedded board, Nvidia Shield K1 tablet	Det: 61%, Rec: 92% (daytime)	Det: 0.026 s, Rec: 0.027 s (Quadro K2200)
Izidio et al. [18]	Custom dataset with 1190 images,	Raspberry Pi3 (ARM Cortex-A53 CPU)	Det: 99.37%, Rec: 99.53% (daytime)	4.88 s
Proposed high-tier solution	CCPD (200,000 images), Synthetic nighttime dataset (CCPD), Real nighttime 100 images	Raspberry Pi 3B+, Intel^®^ NCS2	Det: 90%, Rec: 98.73% (nighttime)	Det: 0.011 s Rec: 0.02176 s

## Data Availability

Not applicable.
